# Efficacy and safety of needle-free injection in patients with type 2 diabetes mellitus undergoing intensive insulin therapy: a randomized controlled trial based on the flash glucose monitoring system

**DOI:** 10.3389/fendo.2025.1652388

**Published:** 2026-01-09

**Authors:** Jing Wang, Zibo Liu, Lijing Jiao, Jing Zhou, Ting Wang, Caige Li, Qian Wang, Lingling Yuan, Fang Zhang, Guizhi Li, Qiuxiao Zhu, Siyao Jin, Lihui Zhang

**Affiliations:** The 2 Hospital of Hebei Medical University, Shijiazhuang, China

**Keywords:** 1,5-anhydroglucitol, blood glucose fluctuation, diabetes mellitus, efficacy and safety, flash glucose monitoring system, intensive insulin therapy, needle-free injector, type 2

## Abstract

This randomized, open-label, parallel-controlled trial investigated the efficacy and safety of needle-free injection (NFI) compared to conventional insulin pen (CIP) in patients with type 2 diabetes mellitus (T2DM) undergoing intensive insulin therapy. A total of 64 hospitalized patients were randomly assigned to receive either NFI or CIP as part of a basal-bolus insulin regimen for 12 ± 2 days. Glycemic parameters were continuously monitored using a flash glucose monitoring (FGM) system. The NFI group showed significantly greater reductions in fasting plasma glucose (FPG) and 2-hour postprandial glucose (2hPPG), along with a more pronounced increase in serum 1,5-anhydroglucitol (1,5-AG) levels compared to the CIP group (all P < 0.05). Glycemic variability was also reduced in the NFI group, as indicated by higher time in range (TIR) and lower mean amplitude (MAGE) and largest amplitude (LAGE) of glycemic excursions (P < 0.05). Participants in the NFI group reached glycemic targets faster than those in the CIP group (1.88 ± 1.36 vs. 2.94 ± 1.59 days, P < 0.05), while daily insulin requirements remained comparable. Furthermore, the NFI group reported fewer injection-site adverse reactions, significantly lower pain scores, and higher treatment satisfaction (P < 0.001), with no increase in hypoglycemic events. These findings suggest that needle-free injection is a safe and effective alternative to conventional insulin delivery in hospitalized patients with T2DM, offering improved glycemic control, reduced glucose variability, and enhanced treatment experience.

**Clinical Trial Registration:**

identifier ChiCTR1900022412.

## 1 Introduction

Diabetes mellitus (DM), a progressive chronic disease marked by hyperglycemia, is growing in prevalence worldwide annually. The International Diabetes Federation reports that there are over 140 million DM patients in China, with over 90% being type 2 diabetes mellitus(T2DM) (1). Persistent metabolic disorders may trigger both acute and chronic complications, profoundly impacting patients’ well-being and mental health, thereby placing a considerable strain on individuals and the healthcare system (2). Intensive insulin therapy is recommended for newly diagnosed T2DM patients exhibiting elevated HbA1c levels ≥9.0%, fasting plasma glucose (FPG) ≥11.1 mmol/L, or pronounced hyperglycemic symptoms. It is also suitable for T2DM patients who, despite undergoing 3 months of oral anti-diabetic medication, continue to have significantly high blood sugar levels (3, 4). This therapeutic approach expedites the mitigation of hyperglycemic toxicity, aids in the preservation of pancreatic β-cell functionality and enhances insulin sensitivity. It ameliorates the metabolic memory effect, retards the emergence and progression of diabetic complications, and thus, enhances patient prognosis (5–8). Intensive insulin treatment is an effective intervention that may potentially reverse T2DM (9).

The basal-bolus insulin regimen effectively mimics the physiological insulin secretion pattern. Its simple dosage adjustment makes it appropriate for most in-hospital intensive treatment scenarios and is widely used in clinical practice. However, the requirement for repeated injections has resulted in some patients delaying or refusing intensive insulin therapy due to needle phobia (10), affecting 3% to 4% of the world’s population and causing fear in 20% to 50% of adolescents (11). Recent study (12) reported that 17.2% of patients are entirely unwilling to initiate insulin therapy, while 34.7% are hesitant. Kim et al. (13) found that 48.2% of patients are reluctant to start insulin treatment due to fear of needles. During insulin therapy, pain and discomfort associated with traditional needle injections, as well as injection-related adverse reactions, can affect treatment adherence, thereby negatively impacting blood glucose control and the quality of life for patients (14, 15).

Needle-free injections, also known as jet injections, provide an innovative solution to the adverse effects of conventional injections (16). The technology applies a pressure jet mechanism that quickly delivers medication into the subcutaneous tissue, ensuring rapid dispersion and mimicking the body’s natural insulin release. Injection depth is consistently 4 to 6 mm, minimizing tissue damage and needle-related risks (17). Endorsed by the Chinese Diabetes Injection Guidelines, these devices are gaining attention for their potential in insulin therapy (18). However, research on their application in intensive insulin therapy for T2DM patients is scarce. This study uses a flash glucose monitoring (FGM) system to assess the efficacy of needle-free injectors.

## 2 Methods

### 2.1 Ethics

The study protocol was approved by the Ethics Committee of the Second Hospital of Hebei Medical University (approval No. 2020-R693-R01) and was conducted in accordance with the Declaration of Helsinki. Written informed consent was obtained from all participants prior to enrollment. This was an exploratory single-center pilot study; therefore, no formal *a priori* sample size calculation was performed, and the planned sample size was determined by feasibility based on the number of eligible hospitalized patients during the recruitment period.

### 2.2 Participants and inclusion/exclusion criteria

This study enrolled hospitalized patients with type 2 diabetes mellitus (T2DM) from the Department of Endocrinology of the Second Hospital of Hebei Medical University. Inclusion criteria were:(1) age between 18 and 70 years; (2) confirmed diagnosis of T2DM; (3) body mass index (BMI) between 18 and 35 kg/m²; (4) for newly diagnosed subjects, hemoglobin A1c (HbA1c) ≥ 9.0% or fasting plasma glucose (FPG) ≥ 11.1 mmol/L; (5) for subjects on a stable regimen of 2–3 oral hypoglycemic agents for ≥ 3 months, persistent HbA1c > 9.0%; (6) ability to communicate clearly with the research team, adhere to study protocols, and provide written informed consent.

Exclusion criteria were: (1) any situation that could result in a conflict of interest with this study; (2) acute or severe chronic diabetes complications, such as recurrent severe hypoglycemia, diabetic ketoacidosis, hyperosmolar coma, diabetic foot, or advanced diabetic nephropathy; (3) severe cardiovascular events within the previous 6 months; (4) current use of systemic glucocorticoids or immunosuppressive drugs, or known immunodeficiency; (5) current use of non-steroidal anti-inflammatory drugs; (6) current treatment with sulfonylureas or other insulin secretagogues; (7) history of malignant tumor; (8) history of unstable or rapidly progressive renal disease; (9) unstable major psychiatric illness; (10) history of hemoglobinopathies such as sickle cell anemia, thalassemia, or severe iron-deficiency anemia; (11) women who were pregnant or breastfeeding; (12) current or anticipated acute infection in the near future (e.g., urinary tract infection, pneumonia); (13) recent major visceral hemorrhage, such as gastrointestinal bleeding or cerebral hemorrhage; (14) active skin diseases at potential injection sites, such as exfoliative dermatitis, pustular lesions, or pyogenic infections; (15) history of acute pancreatitis or pancreatectomy; (16) any other condition that, in the opinion of the investigators, could prevent completion of the study or pose a significant risk to the subject; (17) abnormal laboratory examination results, including:A. liver enzymes > 3 × the upper limit of normal; B. creatinine clearance < 60 mL/min; C. anemia; D. positive pregnancy test in women of childbearing potential.

After admission, all participants were evaluated for diabetes complications according to the 2020 Chinese Diabetes Guidelines. They received structured diabetes education, a standardized diabetic diet, and exercise guidance.

### 2.3 Study design and insulin therapy

**Figure 1:**
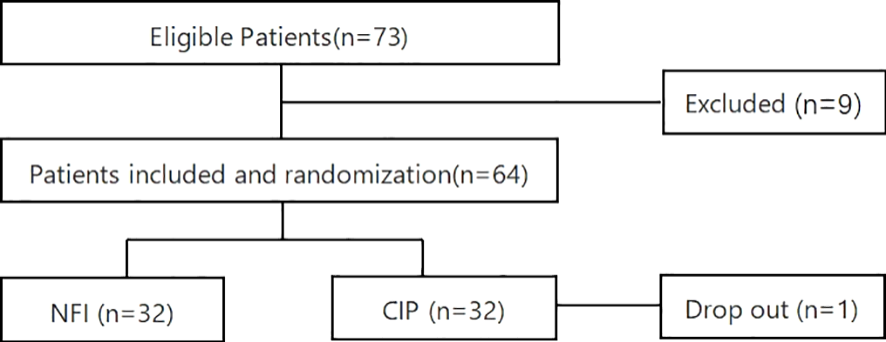
Study design and the flow of patients in the trail. NFI, needle-free injection group; CIP, conventional insulin pen group.

This was a prospective, randomized, open-label, parallel-controlled clinical study designed to evaluate the efficacy and safety of needle-free injectors in hospitalized patients with T2DM treated with an intensive insulin therapy regimen. The trial was conducted in a single tertiary hospital. The study design and patient flow are shown in **Figure 1**.

Participants received intensive insulin therapy following a basal–bolus injection protocol. They were randomly assigned to the needle-free injection (NFI) group or the conventional insulin pen (CIP) group using a random number table. Starting on the day of admission, each participant was equipped with a flash glucose monitoring (FGM) system. Insulin therapy was administered by experienced nurses and adjusted by physicians in accordance with the study protocol. The NFI group used the QS-P needle-free injector (Beijing QS Medical Technology Co., Ltd., China), whereas the CIP group received treatment with the NovoPen 4.0 insulin pen (Novo Nordisk, Denmark). Because of the obvious differences between the injection devices, the study was open label, and neither investigators nor patients were blinded to treatment allocation.

The total initial insulin dose was 0.4 U/kg/day, administered as a basal–bolus regimen with approximately 50% of the total dose given as degludec insulin once daily and 50% as aspart insulin divided before the three main meals. The target fasting plasma glucose (FPG) was 4.4–7.0 mmol/L and the target 2-hour postprandial plasma glucose (2hPPG) was < 10.0 mmol/L. Basal insulin dosage was adjusted every 1–3 days according to FPG (decreased by 2 units if FPG < 4.4 mmol/L, unchanged if 4.4–7.0 mmol/L, increased by 2 units if 7.0–10.0 mmol/L, and by 4 units if > 10.0 mmol/L). Prandial insulin was adjusted according to the postprandial glucose increment (reduced by 2 units if the increase was < 2.0 mmol/L, unchanged if 2.0–4.0 mmol/L, and increased by 2 units if > 4.0 mmol/L). For the outcome “time to reach target blood glucose,” achievement of glycemic target was defined as meeting both the FPG and 2hPPG goals on at least two consecutive days. After the target was achieved, insulin doses continued to be adjusted as needed to maintain glycemic control throughout the remaining observation period.

The observational phase of the study lasted for 12 ± 2 days. Blood samples were obtained at baseline and at the end of the intervention to measure FPG, 2hPPG, 1,5-anhydroglucitol (1,5-AG), and glycated albumin (GA). Glycemic variability indices were derived from FGM data. Clinical characteristics and adverse events were also recorded. After the intervention, a questionnaire survey was administered to participants in both groups to assess injection-site pain and overall treatment satisfaction.

### 2.4 Outcomes and assessment of glucose metrics

The primary outcome was the change from baseline to the end of the study in key glycemic parameters in both groups, including FPG, 2hPPG, GA, and 1,5-AG. For analysis, FPG and 2hPPG were calculated as the mean of three consecutive days at baseline and during the last three days of the intervention, rather than single-point measurements.

Secondary outcomes included:

1. glycemic variability (GV) indicators derived from FGM, namely Time in Range (TIR; percentage of time with glucose levels between 3.9 and 10.0 mmol/L), coefficient of variation (CV) of glucose, standard deviation of blood glucose (SDBG), mean of daily differences (MODD), mean amplitude of glycemic excursions (MAGE), and largest amplitude of glycemic excursions (LAGE). These were evaluated with reference to recommended targets: TIR > 70%, CV < 33%, SDBG < 2.0 mmol/L, MODD < 0.8 mmol/L, MAGE < 3.9 mmol/L, and LAGE < 4.4 mmol/L.
2. Daily insulin dose and the time required to achieve the predefined glycemic targets.
3. Patients’ treatment satisfaction at the end of the trial, assessed using an 11-point numeric rating scale (0–10), where 0 indicated complete dissatisfaction and 10 indicated very high satisfaction with the injection method.

Safety assessments included monitoring of vital signs, the incidence and severity of hypoglycemia, and injection-site reactions (bleeding, bruising, erythema, and swelling). Injection-site pain was evaluated at the end of the observation period using an 11-point numeric rating scale (NRS) ranging from 0 to 10, where 0 indicated no pain and 10 indicated the worst pain imaginable.

### 2.5 Data analysis and statistics

We defined all the participants to the NFI and CIP groups randomly. Demographic information and baseline characteristics were carefully compiled using frequency distributions and descriptive statistics for initial comparisons. Data analysis was conducted using SPSS 21.0. Categorical variables were reported as percentages and analyzed using χ² tests. Continuous variables were described as mean (SD) or median (IQR). Paired t-tests were used for within-group comparisons across different time points. For between-group comparisons at the same time points, normally distributed data were assessed with independent t-tests, and non-normally distributed data were analyzed using the Mann-Whitney U test. The criterion for statistical significance was set at *P*<0.05.

## 3 Results

### 3.1 Basic characteristics of patients

A total of 63 hospitalized patients with T2DM were included in the analysis, with 31 in the CIP group and 32 in the NFI group. Pre-admission glucose-lowering regimens were broadly similar between groups (χ² = 2.102, P = 0.552). In the CIP group, 6.5% of patients were managed with lifestyle measures only, 45.2% with oral antidiabetic drugs (OADs), 16.1% with insulin, and 32.3% with a combination of OADs and insulin. The corresponding proportions in the NFI group were 12.5%, 50.0%, 9.4%, and 28.1%, respectively.

Baseline demographic and clinical characteristics during hospitalization were also comparable between the two groups (**Table 1**). The proportion of male patients was similar (25/6 in CIP vs. 24/8 in NFI, P = 0.590). Mean age was 43.26 ± 13.43 years in the CIP group and 40.72 ± 10.20 years in the NFI group (P = 0.595), and BMI indicated that both groups were overweight (27.34 ± 3.32 vs. 27.01 ± 3.40 kg/m², P = 0.692). The duration of diabetes did not differ significantly between groups, with median values of 8.00 (0.54, 120.00) months in the CIP group and 12.00 (2.04, 36.00) months in the NFI group (P = 0.842). Baseline glycemic control and lipid profiles were similar: FBG (10.99 ± 3.70 vs. 11.43 ± 3.21 mmol/L, P = 0.690), HbA1c (11.11 ± 1.98% vs. 10.74 ± 2.54%, P = 0.118), total cholesterol (4.65 ± 0.85 vs. 4.67 ± 1.21 mmol/L, P = 0.690), and triglycerides (2.85 ± 1.78 vs. 2.26 ± 1.66 mmol/L, P = 0.082). Overall, these findings indicate that pre-admission treatment patterns and baseline characteristics were well balanced between the two treatment groups.

**Table 1:** Baseline characteristics of the patients (n = 63).

Characteristic	CIP (n = 31)	NFI (n = 32)	χ²/Z/t	P
Pre-admission glucose-lowering therapy, n (%)			2.102	0.552
Lifestyle only	2 (6.5)	4 (12.5)		
OADs	14 (45.2)	16 (50.0)		
Insulin	5 (16.1)	3 (9.4)		
OADs + insulin	10 (32.3)	9 (28.1)		
during hospitalization				
Sex (M/F)	25/6	24/8	0.290	0.590
Age (years)	43.26 ± 13.43	40.72 ± 10.20	-0.532	0.595
BMI (kg/m²)	27.34 ± 3.32	27.01 ± 3.40	0.397	0.692
Duration (months)	8.00 (0.54, 120.00)	12.00 (2.04, 36.00)	-0.200	0.842
FBG (mmol/L)	10.99 ± 3.70	11.43 ± 3.21	-0.399	0.690
HbA1c (%)	11.11 ± 1.98	10.74 ± 2.54	-1.561	0.118
TC (mmol/L)	4.65 ± 0.85	4.67 ± 1.21	-0.399	0.690
TG (mmol/L)	2.85 ± 1.78	2.26 ± 1.66	-1.739	0.082

Data are means ± SD.

NFI, needle-free injection group; CIP, the conventional insulin pen group.

BMI, body mass index; DM, diabetes mellitus; FBG, fasting plasma glucose; HbA1c, hemoglobin A1c; TC, total cholesterol; TG, Triglyceride.

### 3.2 Glycemic control indicators

**Figure 2:**
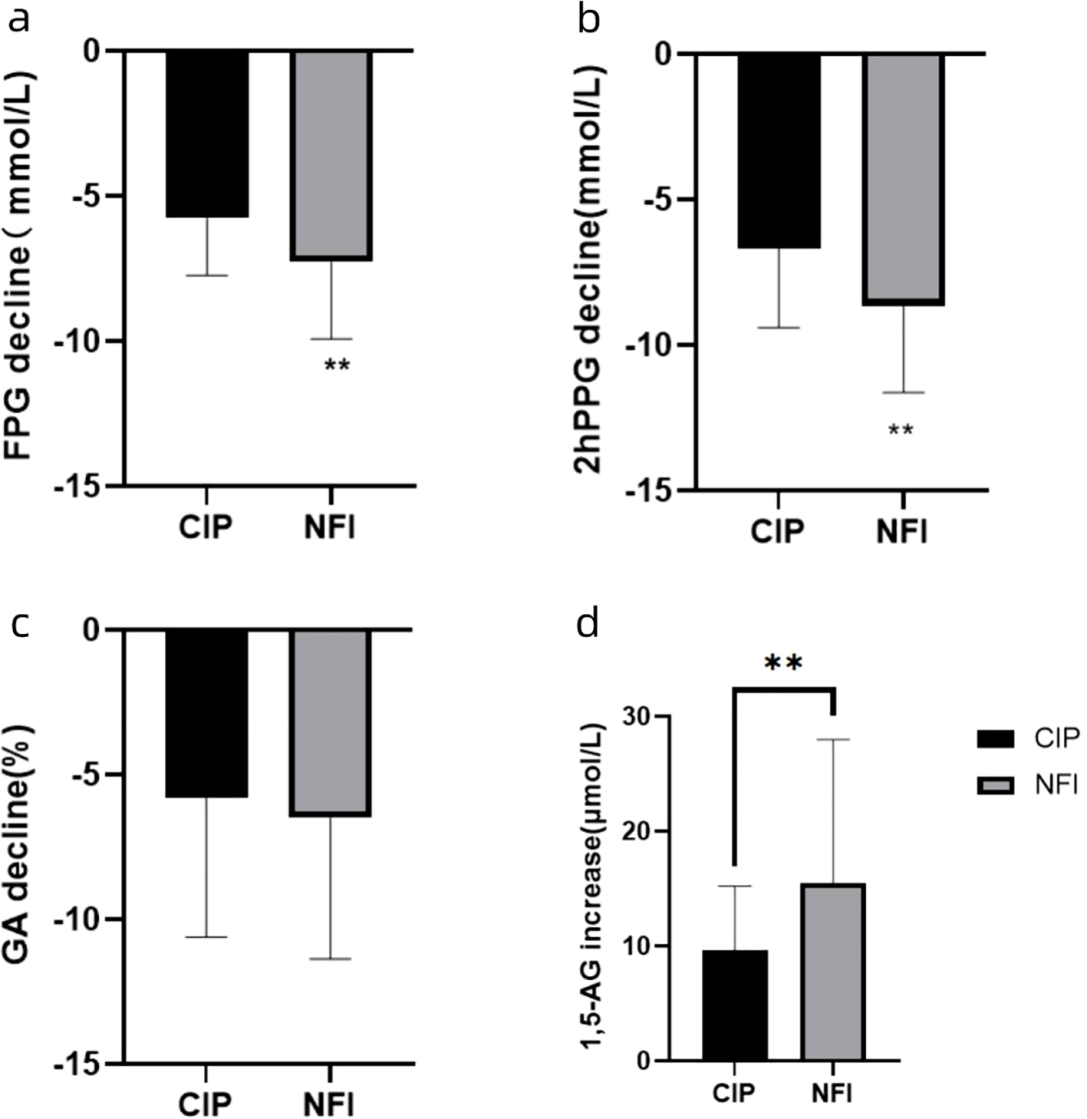
**(A)** FPG decline from baseline in the two groups after 12 ± 2 days’ treatment. Data are presented as mean values ± SEM. **p<0.05 between the two groups. **(B)** 2hPPG decline from baseline in the two groups after 12 ± 2 days’ treatment. Data are presented as mean values ± SEM. **p<0.05 between the two groups. **(C)** GA decline from baseline in the two groups after 12 ± 2 days’ treatment. Data are presented as mean values ± SEM. p>0.05 between the two groups. **(D)** 1,5-AG increase from baseline in the two groups after 12 ± 2 days’ treatment. Data are presented as mean values ± SEM. **p<0.05 between the two groups. NFI, needle-free injection group; CIP, conventional insulin pen group.

Following a 12 ± 2-day intervention, both the NFI and CIP groups demonstrated significant reductions in FPG and 2hPPG from their baseline values. The NFI group saw a more pronounced FPG decline of 7.24 ± 1.65 mmol/L, surpassing the 5.82 ± 1.96 mmol/L observed in the CIP group (*P* < 0.05). The 2hPPG decrease was also more substantial in the NFI group, at 8.65 ± 2.99 mmol/L, compared to 6.68 ± 2.70 mmol/L in the CIP group, marking a statistically significant divergence (*P* < 0.05). Furthermore, the NFI group had a significantly larger increase in 1,5-AG levels, increasing by 15.49 ± 12.51 μmol/L, as opposed to the 9.66 ± 5.58 μmol/L increase in the CIP group, which was statistically significant (*P* < 0.05). Although GA levels decreased for both groups after treatment, the difference between the groups was not statistically significant (*P*>0.05). (**Figure 2**).

### 3.3 Glycemic variability indicators

Glycemic variability indicators were calculated as averages for both groups between days 8 and 10. TIR was higher in the NFI group than in the CIP group (83.62 ± 6.21% vs. 76.83 ± 11.66%), whereas MAGE (2.24 ± 0.86 vs. 2.68 ± 0.81 mmol/L) and LAGE (4.28 ± 1.12 vs. 4.94 ± 1.29 mmol/L) were significantly lower in the NFI group (all P < 0.05). Although SD, MODD, and CV tended to be lower in the NFI group, the differences between the two groups were not statistically significant (P > 0.05; **Table 2**).

**Table 2:** Glycemic variability indicators.

	CIP(n=31)	NFI(n=32)	*Z/t*	*P*
TIR	76.83 ± 11.66	83.62 ± 6.21	-2.911	0.006*
SDBG	2.54 ± 0.56	2.40 ± 0.55	0.997	0.323
MAGE	2.68 ± 0.81	2.24 ± 0.86	-2.418	0.016*
LAGE	4.94 ± 1.29	4.28 ± 1.12	-2.139	0.032*
MODD	2.04 ± 0.56	1.90 ± 0.63	-1.193	0.233
CV	32.58 ± 6.59	29.83 ± 6.44	-1.500	0.134

Data are means ± SD.

NFI, needle-free injection group; CIP, the conventional insulin pen group.

TIR, Time in Range (glucose levels within the range of 3.9 to 10.0 mmol/L); SDBG, Standard Deviation of Blood Glucose; MAGE, Mean Amplitude of Glycemic Excursions; LAGE, Largest Amplitude of Glycemic Excursions; MODD, Mean of Daily Differences; CV, Coefficient of Variation for glucose.

*indicates statistical significance.

**Figure 3:**
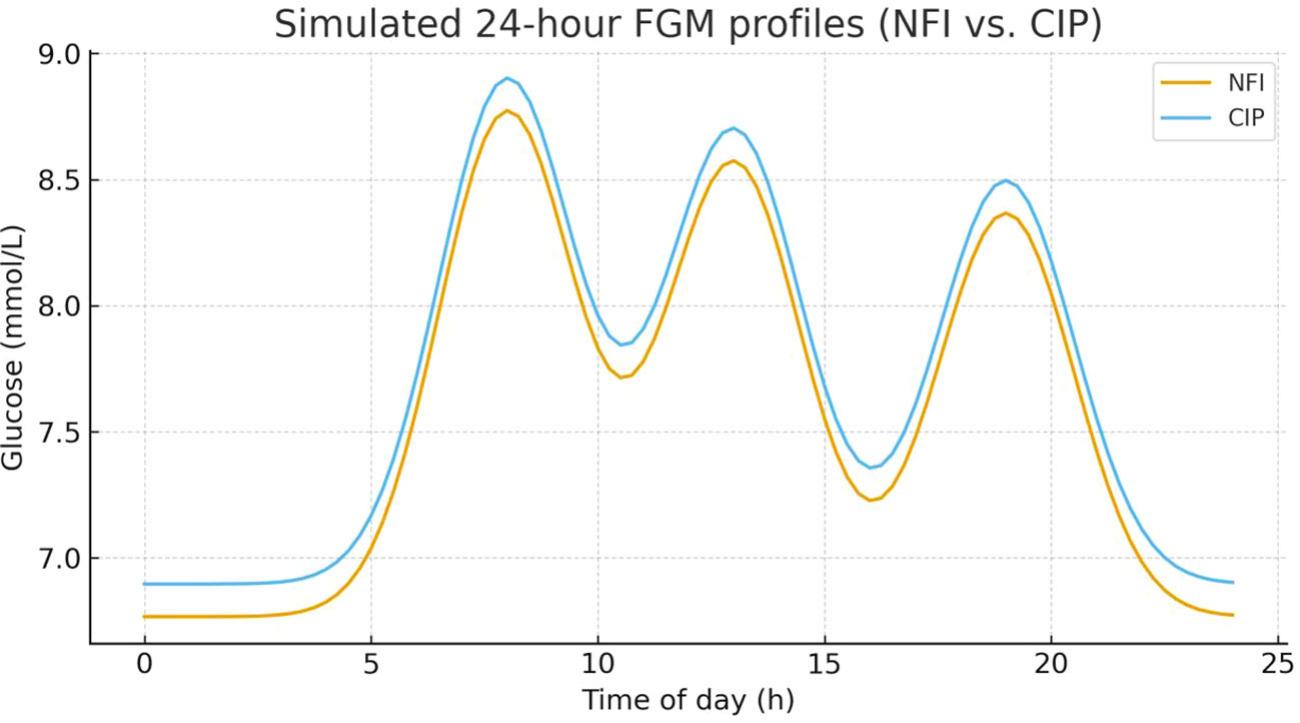
Mean 24-hour flash glucose monitoring (FGM) profiles in the NFI and CIP groups after intensive insulin therapy. Curves represent mean interstitial glucose values over a 24-hour period in the needle-free injection (NFI) group and the conventional insulin pen (CIP) group after 12 ± 2 days of treatment. Both groups show typical daily glycemic patterns with three postprandial peaks. Overall glucose levels and peak amplitudes are lower in the NFI group than in the CIP group. NFI, needle-free injection; CIP, conventional insulin pen.

In addition to these GV indices, 24-hour FGM profiles were used to visualize daily glycemic patterns in the two groups. As illustrated in **Figure 3**, both groups exhibited a similar diurnal pattern characterized by three postprandial glucose peaks. However, the overall glucose curve was slightly lower in the NFI group than in the CIP group throughout the 24-hour period, with lower peak levels after each main meal and marginally lower nocturnal glucose levels.

### 3.4 Daily insulin dosage and time to reach target

**Figure 4:**
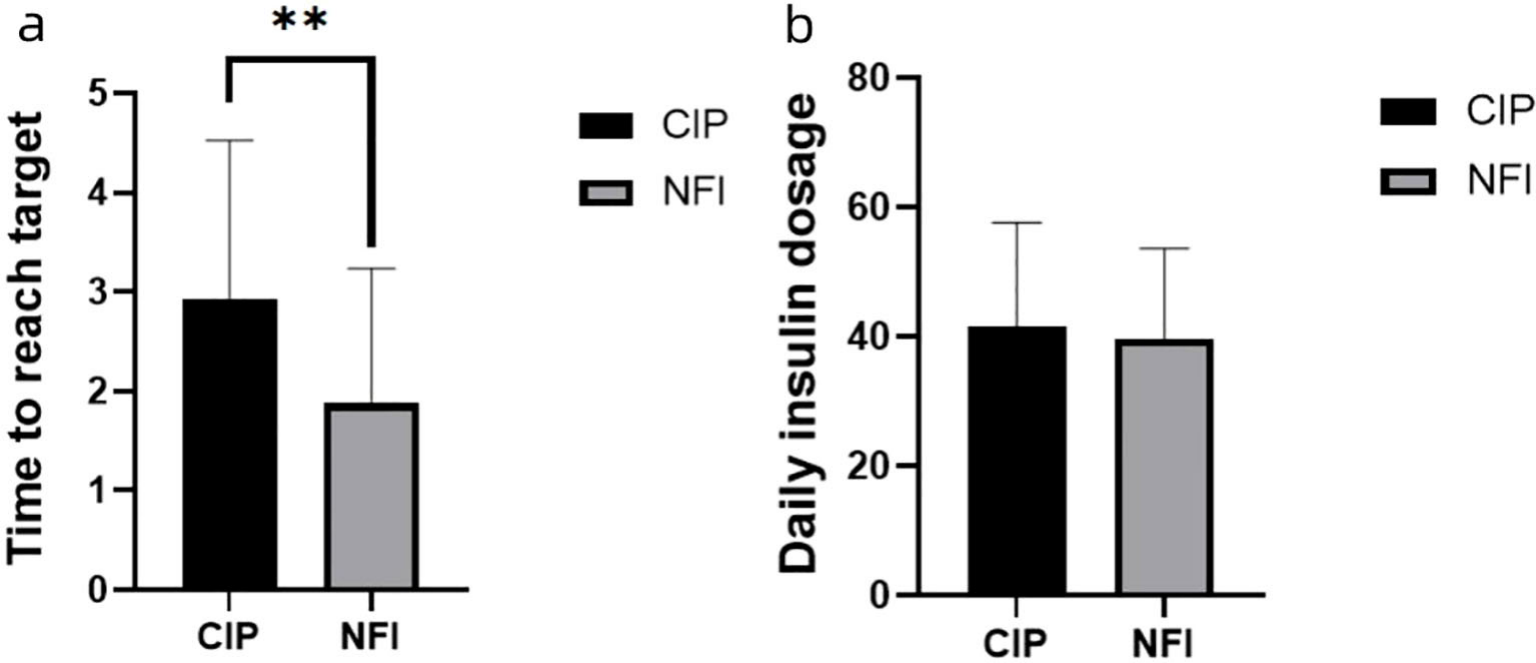
**(A)** Time to reached the target blood glucose levels of the two groups. Data are presented as mean values ± SEM. **p<0.05 between the two groups. **(B)** Daily insulin dosage of the two groups. Data are presented as mean values ± SEM. p>0.05 between the two groups. NFI, needle-free injection group; CIP, conventional insulin pen group.

In both groups, once the glycemic targets were achieved, pre- and postprandial glucose levels were generally maintained within the predefined ranges for the remainder of the study period in most participants. The mean time to reach target blood glucose was significantly shorter in the NFI group than in the CIP group (1.88 ± 1.36 vs. 2.94 ± 1.59 days, P < 0.05), whereas the difference in mean daily insulin dose between the two groups was not statistically significant (P > 0.05; **Figure 4**).

### 3.5 Safety and tolerability

In the safety analysis, hypoglycemia occurred in 4 patients (12.5%) in the NFI group and in 8 patients (25.8%) in the CIP group (P = 0.179). There were no significant differences between the two groups in the rates of mild, moderate, or severe hypoglycemia. Local adverse reactions at the injection site, such as bleeding (12.5% in NFI vs. 25.8% in CIP) and bruising (3.1% in NFI vs. 19.4% in CIP), were less frequent in the NFI group than in the CIP group. Overall skin injury (defined as the composite of bleeding and bruising) was also lower in the NFI group than in the CIP group (18.8% vs. 56.1%; **Table 3**).

**Table 3:** Adverse effects at injection sites.

Group	Bleeding (n)	Bruising (n)	Redness (n)	Swelling (n)	TOTAL (n)
NFI(n=32)	4	1	1	0	6
CIP(n=31)	8	6	1	1	16
χ2	1.808	2.717	/	/	7.483
P	0.179	0.099	0.982	0.492	0.006**

NFI, needle-free injection group; CIP, the conventional insulin pen group.

Number of subjects with adverse effects are shown.

**Figure 5:**
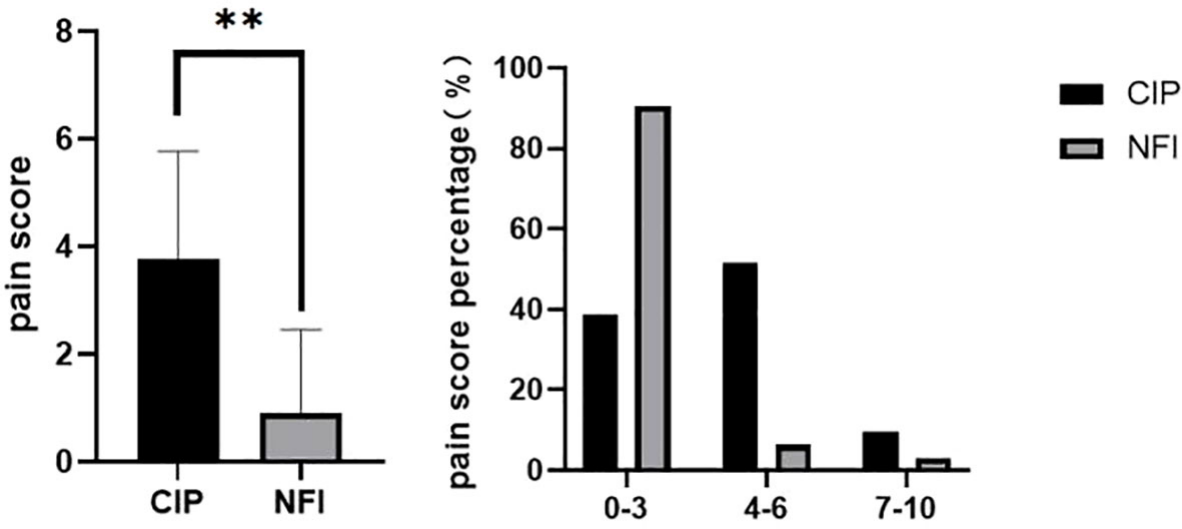
Injection pain scores of the two groups. Data are presented as mean values ± SEM. **P < 0.001. **(B)** Pain score level was stratified into three sections, and patients percentage in each pain score section for NFI and CIP groups are shown. NFI, needle-free injection group; CIP, conventional insulin pen group.

**Figure 6:**
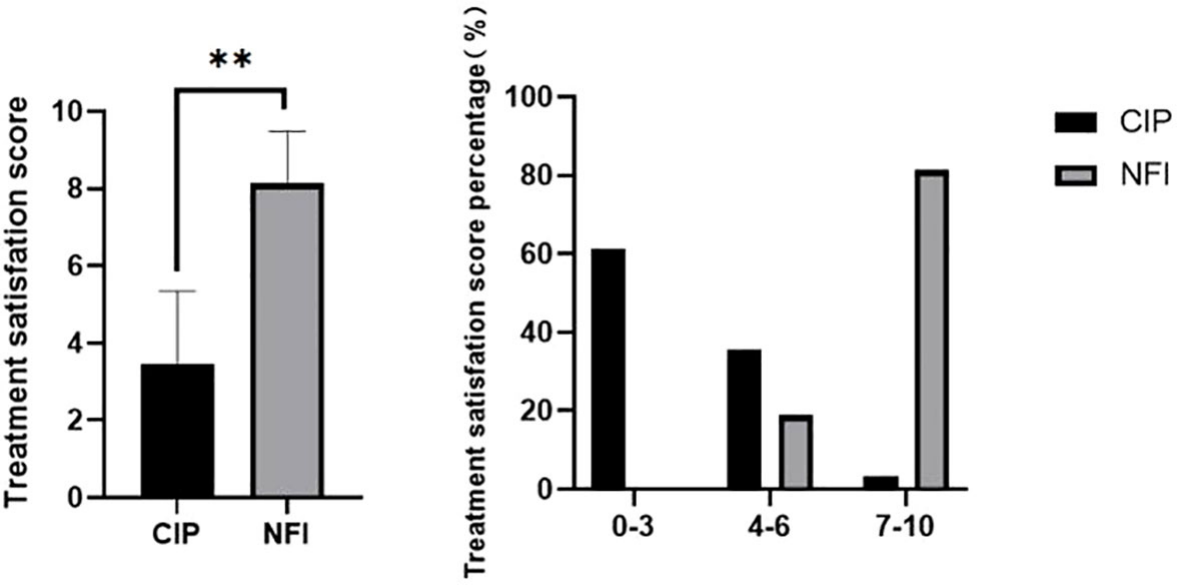
Treatment satisfaction scores in the two groups at the end of the trial. Data are presented as mean values ± SEM. **P < 0.001. Satisfaction scores level was stratified into three sections, and patients percentage in each satisfaction scores section for NFI and CIP groups are shown. NFI, needle-free injection group; CIP, conventional insulin pen group.

Patient-reported outcomes showed marked differences between the two injection methods. Injection-site pain scores were substantially lower in the NFI group than in the CIP group (0.91 ± 1.55 vs. 3.77 ± 1.99; P < 0.001; **Figure 5**). Pain was rated on a 0–10 scale, where 0 indicated no pain, 1–3 mild pain, 4–6 moderate pain, and 7–10 severe pain. Almost 90% of patients in the NFI group had mild pain scores (0–3), whereas pain scores shifted toward the moderate range in the CIP group. Patients also reported greater satisfaction with needle-free injection than with CIP: satisfaction scores were significantly higher in the NFI group (8.13 ± 1.36 vs. 3.45 ± 1.89; P < 0.001; **Figure 6**), with approximately 80% of patients in the NFI group reporting high satisfaction.

## 4 Discussion

The inception of needle-free injection technology traces its roots to the year 1866. Needle-free injectors, when utilized for the delivery of rapid-acting human insulin or insulin analogs, achieve a swifter peak absorption, closely aligning with the natural insulin secretion dynamics (18).This enhanced pharmacokinetic profile translates to improved postprandial blood glucose management and mitigates the discomfort and risks associated with conventional injections, thereby diminishing patient apprehension towards injections. The FREE study’s outcomes (16) underscore the superiority of needle-free injectors over traditional insulin pens in glycemic regulation, without a concurrent rise in hypoglycemic episodes or weight gain. Guo Lixin’s team (20) has illustrated that needle-free administration of rapid-acting insulin analogs facilitates more expeditious absorption, culminating in enhanced blood glucose stabilization within the first 30 minutes postprandially and attenuated glycemic variability.

In this research, participants with T2DM in both the NFI and CIP groups experienced a notable reduction in FBG and 2hPPG relative to baseline values, signifying effective blood sugar reduction through intensive therapy. The NFI group demonstrated a more pronounced decrease in FBG and 2hPPG, with statistically significant disparities between the groups, indicating superior glycemic-lowering efficacy for the NFI method. Both groups exhibited a significant reduction in GA levels from pre-treatment measures; however, no statistically significant difference was observed in the extent of GA reduction between the NFI and CIP groups. GA is an indicator of glycemic control over the preceding 2 to 3 weeks. Given that the study’s observation period was 12 ± 2 days, the brevity of this timeframe may account for the lack of a discernible difference in GA reduction between the two groups.

1,5-AG is a biomarker indicative of glucosuria resulting from hyperglycemia, where its renal reabsorption is subject to competitive inhibition by glucose (21, 22). This characteristic allows for the sensitive detection of acute hyperglycemic changes. 1,5-AG is a robust clinical marker for gauging short-term glycemic management, postprandial glucose spikes, and glycemic fluctuations within a 1 to 2 week timeframe (23, 24). It also serves as an index of β-cell functionality; in individuals with T2DM, diminished 1,5-AG levels exhibit an inverse relationship with FPG and HbA1c levels (25). Research has linked 1,5-AG concentrations to the prevalence of microvascular complications and cardiovascular incidents in T2DM patients (26, 27). Prior to this study, no investigations had explored the relationship between 1,5-AG levels and needle-free injection technology. Our findings illustrate a marked elevation in 1,5-AG levels post-treatment in both NFI and CIP cohorts, indicative of enhanced glycemic fluctuation management in T2DM patients. The NFI group, in particular, displayed a more substantial increase in 1,5-AG levels, with statistically significant disparities from the CIP group, suggesting a superior attenuation of glycemic variability with needle-free injections.

FGM offers an indirect yet continuous reflection of blood glucose levels throughout the day by tracking interstitial fluid glucose. This innovative approach furnishes healthcare providers with an encompassing perspective on glycemic patterns, adeptly delineating the oscillations between hyperglycemia and hypoglycemia, as well as the broader spectrum of glycemic variability. FGM adeptly supplements the inherent constraints of conventional point-of-care glucose testing and HbA1c assessments, thereby amplifying the precision in diagnosing and treating diabetes and ushering in a new era of digital diabetes management (19). Recent study (28–30) has established a substantial negative correlation between TIR for individuals with T2DM and a spectrum of detrimental health outcomes, encompassing cerebral infarction, microvascular complications, diabetic peripheral neuropathy, diabetic ketoacidosis, hyperuricemia, hospitalization expenses, and vertebral fractures.

The MAGE stands as a critical gauge for evaluating day-to-day glycemic fluctuations. The SDBG encapsulates the variability in blood glucose levels, emerging as a more consistent and reliable measure of glycemic stability compared to MAGE. The LAGE identifies the most pronounced single-day glucose swing, and the MODD assesses the extent of variability between consecutive days, reflecting the predictability of glucose levels. Studies (31) have highlighted that SDBG and MAGE are particularly sensitive in detecting glycemic oscillations. Moreover, they posit that fluctuating hyperglycemia, contrasted with persistent hyperglycemia, is more prone to triggering oxidative stress, hastening endothelial cell injury, and thereby exacerbating the incidence and advancement of diabetes-associated complications.

Research conducted by Wu and colleagues (15) has shown that needle-free injection of glargine insulin is particularly effective in T2DM patients with suboptimal FPG levels. This method has been associated with a marked decrease in key glycemic parameters, including the 24-hour mean blood glucose, peak glucose concentrations, the time above range (TAR), MAGE, and SDBG. Additionally, there was a notable enhancement in TIR, which serves to regulate blood glucose levels and their oscillations without increasing the incidence of hypoglycemia.

Our findings reveal that the TIR is higher in the NFI group, while the MAGE and LAGE are considerably lower than in the CIP group. This indicates that needle-free injections may offer superior glycemic control. Despite the needle-free group showing reduced SDBG, MODD, and CV, these reductions did not reach statistical significance, possibly attributed to the limited sample size and the short duration of the intervention in this study. Further research is warranted, with plans to increase the sample size and prolong the intervention period to provide a more definitive analysis.

Research by Ji Qiuhe’s team (32) demonstrated that needle-free injections, in contrast to those administered via insulin pens, decrease the necessary daily insulin dosage to attain desired fasting blood glucose levels by an average of 3.11 units. This reduction is accentuated with higher insulin dosages. Our findings reveal no statistically significant variance in the mean daily insulin dosage between the NFI and CIP groups. Nonetheless, the NFI group reached glycemic targets with greater expedience, averaging 1.88 ± 1.36 days versus 2.94 ± 1.59 days for the CIP group, reflecting a notable disparity. Importantly, the adoption of needle-free injectors enhances the swiftness and efficacy of blood glucose regulation without escalating insulin requirements.

In terms of safety, our findings reveal 4 cases of hypoglycemia within the NFI cohort and 8 within the CIP cohort, without a statistically significant divergence. Importantly, the absence of severe hypoglycemic events in both cohorts implies that needle-free insulin delivery does not elevate the risk of hypoglycemia in the context of short-term intensive insulin therapy for T2DM patients. Furthermore, the deployment of FGM facilitates immediate surveillance of glycemic trends, allowing for prompt insulin dosage modifications and the efficacious avoidance of hypoglycemic episodes.

The FREE study (16) demonstrates that needle-free injectors alleviate the aversion to injections, commonly known as trypanophobia, reduce the sensation of injection pain, and enhance patient satisfaction with treatment. They also mitigate the occurrence of injection-related complications such as lipo-hypertrophy, thereby improving the quality of life for patients undergoing insulin therapy. Recent research (11) indicates that needle-free injectors can ameliorate psychological insulin resistance in T2DM patients.

Our study reveals that the overall incidence of adverse reactions at the injection site in the needle-free group is significantly lower than in the CIP group, with NFI group also exhibiting significantly lower pain scores and markedly higher patient satisfaction scores. The utilization of needle-free injectors can elevate patient treatment satisfaction, diminish the psychological stress and physical discomfort associated with injections, and thus enhance the overall quality of life for patients.

Needle-free injectors may hold potential clinical applications in various domains: the glycemic management of type 1 diabetes, gestational diabetes, and the treatment of pediatric and geriatric patients with diabetes. They could also serve as a delivery method for other anti-diabetic injectables such as glucagon-like peptide-1 receptor agonists (GLP-1RA), combination injectable formulations (like degludec liraglutide injection), and dual GLP-1/GIP agonists. These applications could significantly enhance patient therapeutic experiences and quality of life. There is an expectation that needle-free injectors will play an increasingly pivotal role in future diabetes management. However, further research is essential to ascertain the long-term efficacy, cost-effectiveness, and patient acceptance of these devices.

This study has several limitations. First, the intervention period was relatively brief (12 ± 2 days) and the study was conducted entirely in an in-hospital setting. Moreover, the duration of FGM use was shorter than the at least 14 days recommended by the international consensus on CGM, and thus our findings regarding sensor-derived glucose metrics should be considered exploratory and short term. Second, there was a gender imbalance, with a predominance of male patients, which may limit the generalizability of the results. Third, potential measurement errors associated with FGM were not explicitly taken into account, which could have influenced the assessment of blood glucose and glycemic variability. Fourth, we did not perform systematic follow-up after completion of the study, and therefore could not evaluate the persistence of glycemic benefits, device acceptance, or patient-reported outcomes in the longer term. Fifth, this was an exploratory single-center pilot study without a formal *a priori* sample size calculation, and the relatively small sample size may limit both the statistical power and the generalizability of our findings. Finally, pain and treatment satisfaction were assessed using simple numeric rating scales rather than disease-specific validated questionnaires, and standardized measures of adherence and treatment attitudes were not applied; this may limit the precision and comparability of these subjective outcomes across studies. Further multicenter trials with larger samples, longer follow-up, and comprehensive validated PROMs are warranted to confirm and extend our observations.

In addition to short-term improvements in glycemic control, our study highlights the potential benefits of needle-free injection on patient-reported outcomes. Patients treated with NFI reported substantially less injection-site pain and greater overall satisfaction with their insulin regimen than those using conventional insulin pens. These differences may be clinically relevant, as comfort and acceptance of insulin therapy are key determinants of long-term adherence in people with T2DM. Although our observation period was limited to the in-hospital setting, the favorable patient experience with NFI suggests that this approach might help overcome psychological barriers to insulin treatment, particularly in individuals with needle anxiety or poor treatment acceptance. Future studies with longer follow-up and PROMs as primary endpoints are needed to confirm these findings and to evaluate whether improved patient experience translates into better long-term glycemic control.

The study also showed a very high completion rate, with all participants in the NFI group completing the trial and only one dropout in the CIP group due to needle-related anxiety, supporting the overall integrity of the data. In summary, the use of needle-free injection technology in intensive insulin therapy for hospitalized patients with T2DM was associated with effective short-term glycemic control, a shorter time to achievement of glycemic targets, and reduced glycemic variability, without an increased risk of hypoglycemia. Furthermore, NFI alleviated fear of needles and reduced local injection-related adverse reactions, offering a potentially more acceptable mode of insulin administration for patients with T2DM.

## Data Availability

The original contributions presented in the study are included in the article/supplementary material. Further inquiries can be directed to the corresponding author.
